# Honey-Induced Protein Stabilization as Studied by Fluorescein Isothiocyanate Fluorescence

**DOI:** 10.1155/2013/981902

**Published:** 2013-10-09

**Authors:** Yin How Wong, Habsah Abdul Kadir, Saad Tayyab

**Affiliations:** Biomolecular Research Group, Biochemistry Programme, Institute of Biological Sciences, Faculty of Science, University of Malaya, 50603 Kuala Lumpur, Malaysia

## Abstract

Protein stabilizing potential of honey was studied on a model protein, bovine serum albumin (BSA), using extrinsic fluorescence of fluorescein isothiocyanate (FITC) as the probe. BSA was labelled with FITC using chemical coupling, and urea and thermal denaturation studies were performed on FITC-labelled BSA (FITC-BSA) both in the absence and presence of 10% and 20% (w/v) honey using FITC fluorescence at 522 nm upon excitation at 495 nm. There was an increase in the FITC fluorescence intensity upon increasing urea concentration or temperature, suggesting protein denaturation. The results from urea and thermal denaturation studies showed increased stability of protein in the presence of honey as reflected from the shift in the transition curve along with the start point and the midpoint of the transition towards higher urea concentration/temperature. Furthermore, the increase in Δ*G*_*D*_^H_2_O^ and Δ*G*_*D*_^25°C^ in presence of honey also suggested protein stabilization.

## 1. Introduction

Protein stability has been regarded as a critical issue in biotechnology due to increasing demands of proteins in various applications, such as industrial enzymes, analytical tools, therapeutics agents, clinical diagnostic materials, and so forth [1–3]. However, many proteins suffer from the drawback of poor storage and operational stability and lose their functionality and native conformation over a period of time [1]. On the other hand, various industrial processes require operational conditions such as extreme temperature, pH, pressure, and the presence of various chemicals and solvents, which may lead to protein destabilization and denaturation. Hence, there is a need to increase the stability of proteins used in industrial processes. 

 Several attempts have been made to increase protein stability using chemical modification, site-directed mutagenesis, introduction of disulphide bonds, and anion binding sites as well as solvent engineering [4–7]. However, only a few modified enzymes have displayed commercial viability [8, 9]. Solvent engineering has shown an edge over other methods of protein stabilization as osmolytes are known to be functionally and biologically compatible with most of the proteins [2, 10, 11]. Sugars and amino acids are well-known naturally occurring osmolytes, showing great protein stabilizing potential against different denaturing conditions including chemical (urea and guanidine hydrochloride) and thermal denaturations [1, 12–14]. With a few exceptions [12, 15, 16], most of the osmolyte-induced protein stabilization studies have been focused on using a single osmolyte [3, 13, 14].

Honey is a natural mixture of compounds with high-sugar content (~38% fructose, ~32% glucose, ~1.7% maltose, ~1.4% sucrose, ~1% turanose, ~0.8% isomaltose, ~0.4% erlose, etc.) and minor constituents such as amino acids, proteins, vitamins, minerals, and polyphenols [17, 18]. Due to the presence of these compounds, honey has been regarded as the traditional medicine and food supplement since prehistorical time. Modern science has also recognized the antibacterial, antioxidant, anti-inflammatory, and wound healing properties of honey [18]. Due to the presence of a number of osmolytes, honey can be used as a potential protein stabilizer. Recently, an attempt has been made to study protein stabilizing potential of honey using simulated honey sugar cocktail with sugar composition similar to that found in honey [16]. Our preliminary studies (unpublished observations) have suggested that the protein stabilization studies in the presence of honey using fluorescence spectroscopy are hampered due to interference by the presence of proteins as well as other molecules in honey, which act as fluorescence quenchers. Therefore, fluorescein isothiocyanate (FITC) fluorescence has been used to study protein stabilization against urea and thermal denaturations in the presence of honey using FITC fluorescence spectroscopic signal after labelling the model protein, bovine serum albumin (BSA), with FITC. 

## 2. Materials and Methods

### 2.1. Materials

Bovine serum albumin, essentially fatty acid free (type A-6003), fluorescein isothiocyanate, isomer I (type F-7250), and urea ultrapure (type U-0631) were purchased from Sigma-Aldrich, Inc., USA. Unblended clover honey from New Zealand (Batch 120708) was purchased from Caremark SDN BHD, Malaysia. All other reagents used were of analytical grade purity.

### 2.2. FITC Labelling of BSA

The protein solution (10 mg BSA/mL in 100 mM sodium carbonate buffer, pH 9.0) was titrated with increasing volumes of FITC (20 mg/mL in absolute ethanol) to achieve an FITC/BSA molar ratio of 20 : 1. The reaction mixture was stirred overnight in dark at 4°C, followed by dialysis against 60 mM sodium phosphate buffer, pH 7.4, to remove unconjugated FITC. The dialysis was continued with several changes of the buffer until the light absorption of the dialysate at 495 nm reached a value <0.003. The FITC-labelled BSA (FITC-BSA) was concentrated to a final concentration of 13.34 mg/mL and stored in dark at 4°C.

### 2.3. Analytical Procedures

The concentration of FITC-labelled BSA as well as FITC to protein molar ratio (F : P molar ratio) was obtained in the same way as described by Hungerford et al. [19]. The concentration of stock urea solution was determined by the method of Pace and Scholtz [20].

### 2.4. Preparation of Stock Honey and Urea Solutions

Stock honey solution (60%, w/v) was prepared by dissolving 600 g of honey in 60 mM sodium phosphate buffer, pH 7.4 in a total volume of 1000 mL, and the pH of the solution was adjusted to pH 7.4 with sodium hydroxide. The stock honey solution was diluted with the above buffer to get the desired honey concentrations, and the solutions were filtered through 0.45 *μ*M Millipore filter before use to remove the water-insoluble components of honey such as wax, pollen, honey comb debris, filth particles, and ash [21].

 Stock urea solutions (11.0 M), both in the absence and presence of honey, were prepared by dissolving 67 g of urea in 60 mM sodium phosphate buffer, pH 7.4, with or without the desired honey concentration to a final volume of 100 mL.

### 2.5. Fluorescence Spectroscopy

The fluorescence spectra of FITC-BSA were obtained on a Jasco spectrofluorometer, model FP-6500, using a 1 cm path length cell with a protein concentration of 0.5 *μ*M at 25°C (unless otherwise stated) upon excitation at 495 nm. The emission spectra were recorded between 500 nm and 600 nm and fluorescence signals obtained were corrected using the corresponding honey solutions, prepared in the same way except that buffer was used instead of the protein solution.

### 2.6. Urea Denaturation

Stock FITC-BSA solution (200 *μ*M) was diluted to 20 *μ*M with desired volume of stock honey solution or buffer to get a desired concentration of honey. For urea denaturation experiments, different volumes of the buffer were added first to tubes containing a fixed volume (125 *μ*L) of the protein solution (20 *μ*M FITC-BSA) followed by the addition of different volumes of the stock urea solution to obtain the desired concentration of urea. The total volume of the solution mixture was 5 mL and the final concentration of FITC-BSA was 0.5 *μ*M. The tubes were shaken well and incubated for 12 hours in dark at 25°C before spectral measurements. Experiments involving honey were carried out in the same way except that all solutions contained the desired concentration of honey. Each experiment was repeated at least three times and the difference in the reported analytical values was found to lie within ±3%.

 Data obtained with FITC fluorescence was transformed into the fraction denatured, *F*_*D*_, using the equation

(1) $$\begin{array}{c} F_{D}=\frac{Y-Y_{N}}{Y_{D}-Y_{N}}, \end{array}$$

where *Y* represents the observed variable parameter at a given denaturant concentration and *Y*_*N*_ and *Y*_*D*_ are the values of the variable characteristic of the native and denatured states, respectively [20]. Values of *Y*_*N*_ and *Y*_*D*_ were obtained by linear extrapolation of pre- and post-transition zones of the denaturation curve. Values of equilibrium constant, *K*_*D*_, and free energy of denaturation, Δ*G*_*D*_, were calculated following equations (2) and (3), respectively:

(2) $$\begin{array}{c} K_{D}=\frac{F_{D}}{1-F_{D}}, \end{array}$$

(3) $$\begin{array}{c} \Delta G_{D}=-RT\;\mathrm{In}K_{D}, \end{array}$$

where *R* is the gas constant (1.987 cal mol^−1 ^K^−1^) and *T* is the absolute temperature in K. The free energy of stabilization (Δ*G*_*D*_^H_2_O^) with or without different honey concentrations in the absence of urea was obtained from the *Y*-intercept of Δ*G*_*D*_ versus urea concentration plot using least squares analysis, whereas the midpoint of denaturation (*C*_*m*_) was calculated from the linear regression equation [20]. 

### 2.7. Thermal Denaturation

Thermal denaturation studies were carried out on a Jasco spectrofluorometer, model FP-6500 equipped with a Protech 632D temperature-controlled circulating water bath. The fluorescence spectra of FITC-BSA both in the absence and presence of desired honey concentrations (10% and 20%, w/v) were recorded in the wavelength range of 500–600 nm upon excitation at 495 nm in the temperature range of 25−100°C after equilibrating the protein sample for 6 min at each temperature. The denaturation data were transformed into fraction denatured (*F*_*D*_) using equation (1) and analysed using a two-state model as described elsewhere [20].

## 3. Results and Discussions

### 3.1. Influence of Honey on FITC-BSA Fluorescence Spectra

Preliminary studies on the fluorimetric investigation of protein stability in the presence of honey have shown complete quenching of the protein's intrinsic fluorescence in the presence of honey (unpublished data). Such quenching of the protein's intrinsic fluorescence can be ascribed to the presence of various compounds such as flavonoids and polyphenols in the honey, which could act as the quenchers of protein's fluorescence. In view of the unsuitability of intrinsic fluorescence probe to study protein stabilization in the presence of honey, FITC was chosen as an extrinsic fluorescence probe for such studies as both the excitation and emission wavelengths were different from the protein's intrinsic fluorescence excitation and emission wavelengths. Hence, the model protein, BSA, was labelled with FITC following the procedure described in Section 2 and used in the urea and thermal denaturation studies both in the absence and presence of 10% and 20% (w/v) honey. It is important to note that FITC was chemically coupled to the *ε*-amino group of lysine residues in the FITC-labelled protein molecule [22]. 

**Figure 1:**
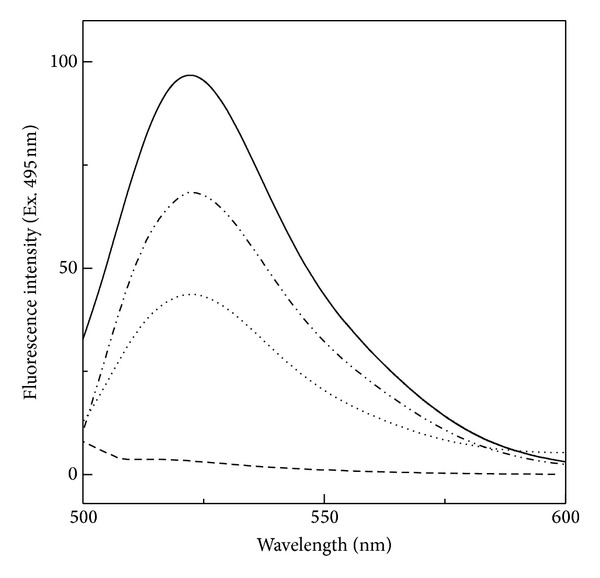
Fluorescence spectra of 0.50 *μ*M FITC-BSA both in the absence (—) and presence of 10% (– *·* *·* –) and 20% (*·* *·* *·* *·*) (w/v) honey upon excitation at 495 nm. Fluorescence spectrum of 0.5 *μ*M BSA (- - -) is also shown.

 The fluorescence spectrum of FITC-BSA was characterized by the presence of a single emission maxima at 522 nm, spectral characteristic of FITC group (Figure 1). On the other hand, unlabelled BSA did not produce any fluorescence signal within this wavelength range, when excited at 495 nm (Figure 1). These results suggested that the fluorescence spectrum produced by FITC-BSA was solely due to the presence of FITC group in the labelled BSA without any interference of the protein's intrinsic fluorescence. Hence, FITC fluorescence can be used as a probe to study protein stabilization of a labelled protein in the presence of other proteins in the system. Although a decrease in the fluorescence intensity was observed at the emission maxima in the presence of 10% and 20% (w/v) honey (Figure 1), it was far lesser than the decrease observed in the intrinsic fluorescence showing complete quenching in the presence of ~5% (w/v) honey (unpublished data).

### 3.2. Urea Denaturation of FITC-BSA in the Absence and the Presence of Honey

**Figure 2:**
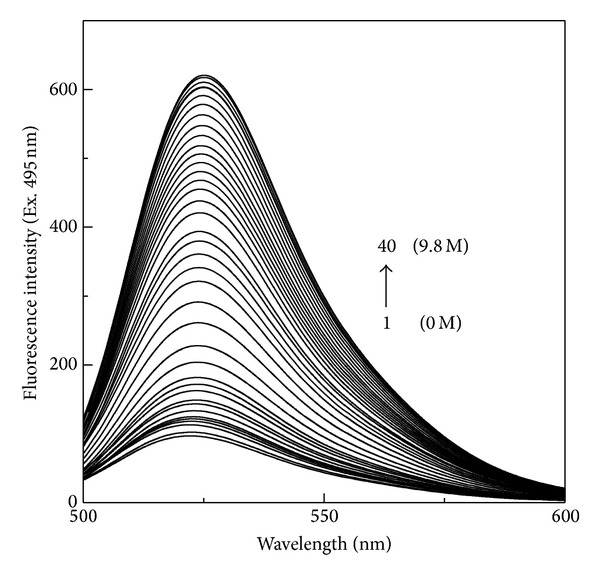
Fluorescence spectra of 0.50 *μ*M FITC-BSA in the absence and presence of increasing urea concentrations upon excitation at 495 nm. The urea concentrations (1 → 40) were 0, 0.5, 1.0, 1.5, 2.0, 2.5, 3.0, 3.5, 3.8, 4.0, 4.2, 4.4, 4.6, 4.75, 4.8, 5.0, 5.2, 5.4, 5.6, 5.8, 6.0, 6.2, 6.4, 6.6, 6.8, 7.0, 7.2, 7.4 7.6, 7.8, 8.0, 8.25, 8.5, 8.75, 9.0, 9.3, 9.4, 9.5, 9.7, and 9.8 M.

Figure 2 shows the fluorescence spectra of FITC-BSA both in the absence and presence of increasing urea concentrations. As can be seen from this figure, addition of increasing urea concentrations to FITC-BSA produced a progressive increase in the fluorescence intensity accompanied by a small red shift in the emission maxima. About 6.3-fold increase in the fluorescence intensity at 522 nm along with a 3 nm red shift in the emission maxima was observed in the presence of 9.8 M urea. Increase in the fluorescence intensity at 522 nm and the red shift in the emission maxima have also been shown earlier with FITC-BSA in the presence of 6.5 M urea [19]. Since FITC was chemically linked to the lysine residues in BSA molecule; hence, in FITC-BSA, several FITC molecules must be in close proximity with each other in the globular three-dimensional structure as a significant number of lysine pairs were found separated within 20 Å in the cross-linking and mass spectrometry studies on BSA [23]. Thus, the formation of nonfluorescent dimers due to the association of FITC molecules can account for the concentration quenching in FITC-BSA [24, 25]. Furthermore, resonance energy transfer from excited FITC molecules to a nonfluorescent dimer might have caused further quenching in the fluorescence. Additionally, a small Stokes' shift in fluorescein molecule might have allowed homoenergy transfer [26]. Thus, FITC-BSA seems to be a highly quenched molecule. In view of this, increase in the fluorescence intensity of FITC-BSA upon addition of urea can be explained on the basis of the phenomenon of fluorescence dequenching. Since, urea removes all noncovalent interactions known to stabilize the protein globular structure, transformation of globular structure into a cross-linked random coil in the presence of urea would have separated the proximal FITC groups away from each other and thus caused the release in fluorescence quenching (fluorescence dequenching). Such a release in fluorescence quenching would have been responsible for the increase in the fluorescence intensity observed in the presence of urea. Separation of FITC groups in the urea-denatured protein can also explain the small red shift of 3 nm in the emission maxima (from 522 nm to 525 nm) observed in the presence of 9.8 M urea. This seems understandable as free FITC is known to display emission maxima at 525 nm in its fluorescence spectrum [27]. Occurrence of a red shift of 3 nm in the fluorescence spectra of FITC-BSA in the presence of 9.8 M urea suggested that the microenvironment around the FITC molecule became more polar than that in the native state.

**Figure 3:**
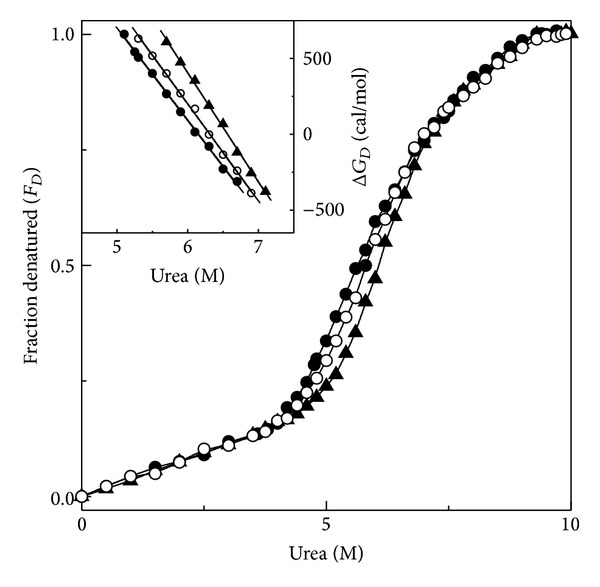
Normalized transition curves of urea-induced denaturation of FITC-BSA both in the absence (*⚫*) and presence of 10% (○) and 20% (▲) (w/v) honey as monitored by FITC fluorescence at 522 nm upon excitation at 495 nm. Inset shows dependence of Δ*G*_*D*_ of FITC-BSA on urea concentration in the absence (*⚫*) and presence of 10% (○) and 20% (▲) (w/v) honey.

 Transformation of the fluorescence intensity values at 522 nm into fraction denatured, *F*_*D*_, at different urea concentrations yielded the denaturation curve shown in Figure 3. Data obtained from urea denaturation experiments of FITC-BSA performed in the presence of 10% and 20% (w/v) honey concentrations were transformed in the same way and are also included in Figure 3. The increase in the fluorescence intensity of FITC-BSA upon the addition of urea was found to be smaller at lower urea concentration range (0.25–4.0 M urea), which was suggestive of small structural perturbation in FITC-BSA within this urea concentration range. A marked increase in the fluorescence intensity was observed between 4.0 M and 9.3 M urea, indicating major structural alteration in the protein beyond which no significant change in the fluorescence intensity was observed, suggesting completion of the denaturation phenomenon. As can be clearly seen from Figure 3, urea denaturation of FITC-BSA followed a single-step, two-state transition with the start point and the end point occurring at ~4.0 M and ~9.3 M urea, respectively (Figure 3 and Table 1). The start point observed with FITC-BSA was found to be relatively higher compared to the start point obtained in the urea denaturation curve of the unlabelled BSA [16], suggesting greater stability of FITC-BSA than unlabelled BSA. However, this change in the stability of FITC-BSA will not affect our conclusions about honey-induced stabilization since the stability of FITC-BSA was compared both in the absence and presence of honey. Although urea-induced denaturation of unlabelled BSA showed a two-step, three-state transition with the accumulation of an intermediate state when monitored by intrinsic fluorescence measurements [16, 28], the absence of intermediate and single-step, two-state transition observed with FITC fluorescence probe simply reflects the different searching mechanisms of protein denaturation by different probes. Use of several other probes such as 8-anilino-1-naphthalene sulfonate (ANS) fluorescence and tryptophan fluorescence also showed a single-step, two-state transition of urea denaturation of serum albumin [29–31]. Yet in another study on urea denaturation of BSA in the presence of 1.0 M KCl, a single-step, two-state transition was observed using intrinsic fluorescence as the probe [28]. Therefore, the nature of the transition curve of urea denaturation depends on the probe used.

 Urea-induced denaturation curves of FITC-BSA obtained in the presence of 10% and 20% (w/v) honey concentrations were qualitatively similar to that obtained in the absence of honey. However, the transition zone was shifted towards higher urea concentration showing increment in both the start point and the midpoint of transition, suggesting protein stabilization in the presence of different honey concentrations (Figure 3 and Table 1). This shift in the transition curve was found to be concentration dependent, being more pronounced in the presence of 20% (w/v) honey concentration. More evidently, the values of the start point and the midpoint of the transition in the presence of 20% (w/v) honey were 4.4 M and 6.1 M compared to 4.2 M and 5.8 M, obtained in the presence of 10% (w/v) honey (Table 1). Quantitative analysis of the protein stabilization studies in the presence of honey can be made by determining the values of the free energy of stabilization of the protein, Δ*G*_*D*_^H_2_O^, from the Δ*G*_*D*_ versus urea concentration plots, shown in the inset of Figure 3, and these values are listed in Table 1. Δ*G*_*D*_^H_2_O^ of FITC-BSA was found to be 3400 cal/mol and increased (29%) to 4400 cal/mol in the presence of 20% (w/v) honey that further supported the protein stabilization in the presence of honey. Both the shifts in the transition curve, characterized by the increase in the start point and the midpoint of denaturation and increase in the Δ*G*_*D*_^H_2_O^ value, obtained in the presence of 10% and 20% (w/v) honey, were indicative of protein stabilization of FITC-BSA in the presence of honey.

### 3.3. Thermal Denaturation of FITC-BSA in the Absence and the Presence of Honey

**Figure 4:**
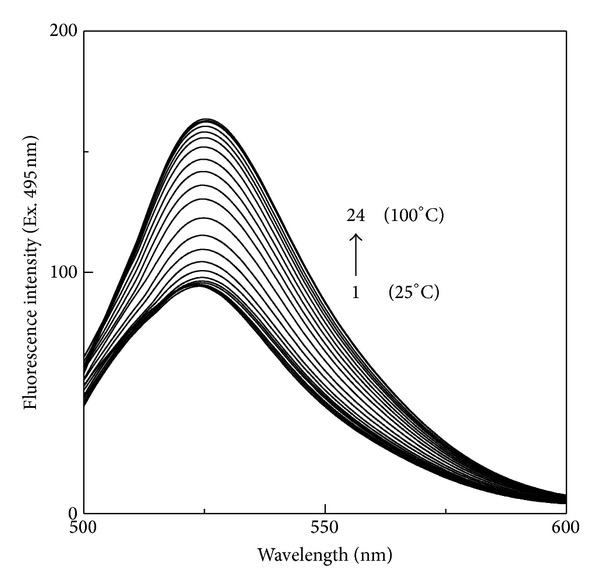
Fluorescence spectra of 0.50 *μ*M FITC-BSA obtained at increasing temperatures in the range of 25–100°C upon excitation at 495 nm. Different temperatures (1 → 24) used were: 25, 30, 35, 40, 45, 50, 52.5, 55, 57.5, 60, 62.5, 65, 67.5, 70, 72.5, 75, 77.5, 80, 82.5, 85, 90, 95, 97.5 and 100°C.

**Figure 5:**
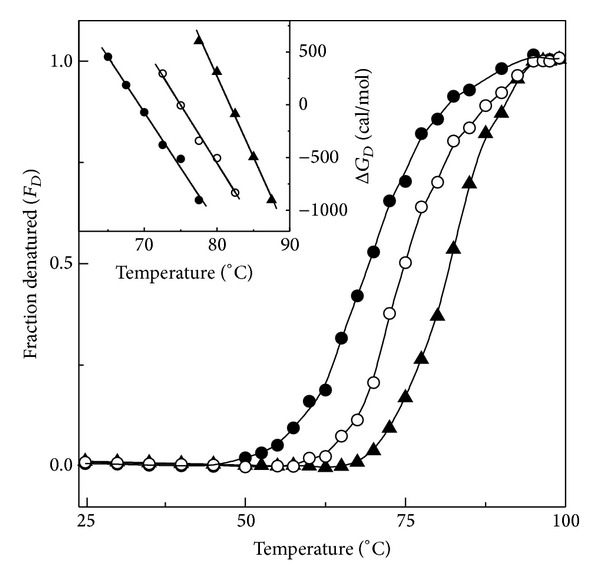
Normalized transition curves of thermal-induced denaturation of FITC-BSA both in the absence (*⚫*) and presence of 10% (○) and 20% (▲) (w/v) honey as monitored by FITC fluorescence at 522 nm upon excitation at 495 nm. Inset shows dependence of Δ*G*_*D*_ of FITC-BSA on temperature in the absence (*⚫*) and presence of 10% (○) and 20% (▲) (w/v) honey.

In order to study the thermal stabilizing potential of honey, thermal denaturation studies of FITC-BSA both in the absence and presence of different honey concentrations (10% and 20%, w/v) were performed.  Figure 4 shows the fluorescence spectra of FITC-BSA, obtained at different temperatures within the temperature range of 25–100°C. Similar to the results obtained with urea denaturation studies of FITC-BSA (Figure 2), the increase in temperature also led to an increase in the fluorescence intensity and a small red shift in the emission maxima. Values of the fluorescence intensity at 522 nm obtained at different temperatures were transformed into fraction denatured, *F*_*D*_, as described in Section 2 and are plotted against temperature (Figure 5). Thermal transition curve of FITC-BSA showed no change in the fluorescence intensity in the initial temperature range (25–45°C), a marked increase within the temperature range (45–95°C), and no change in the fluorescence intensity beyond 95°C. Fluorescence data obtained in the presence of 10% and 20% (w/v) honey were transformed in the same way and are shown in Figure 5. Thermal denaturation of FITC-BSA showed a single-step, two-state transition with the occurrence of the start point and the end point at 45°C and 95°C, respectively (Figure 5). The midpoint (melting temperature, *T*_*m*_) was observed at 69.3°C. Thermal transition curve of FITC-BSA obtained in the presence of 10% and 20% (w/v) honey showed a marked shift in the transition curve towards higher temperature. Values of the start point, midpoint, and end point obtained in the thermal transition curve of FITC-BSA in the presence of different honey concentrations are given in Table 2. Both start point and midpoint showed an increase in the presence of honey, being higher at 20% (w/v) honey concentration (Table 2). Both the shift in the transition curve and increase in the start point and midpoint values of FITC-BSA thermal transition curve observed in the presence of 10% and 20% (w/v) honey were suggestive of honey-induced FITC-BSA stabilization against temperature. 

**Figure 6:**
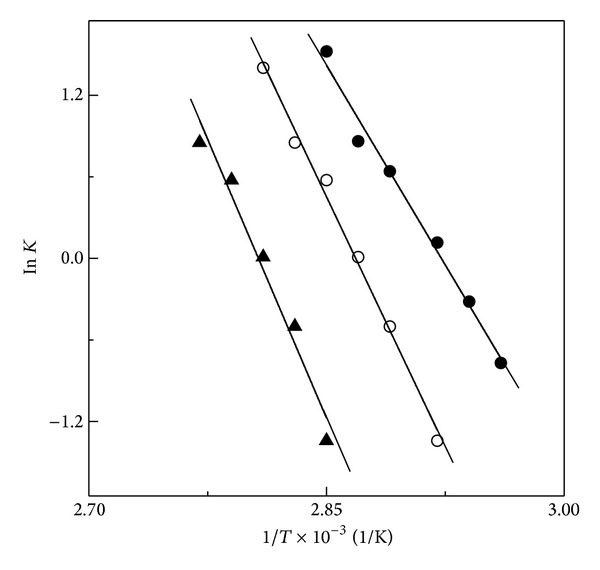
van't Hoff plot of thermal denaturation results of FITC-BSA both in the absence (*⚫*) and presence of 10% (○) and 20% (▲) (w/v) honey, as obtained from the results shown in Figure 5.

 Determination of Δ*G*_*D*_^25°C^ was made using Δ*G*_*D*_ versus temperature plots (inset of Figure 5) both in the absence and presence of different honey concentrations. As can be seen from Table 2, the presence of 10% and 20% (w/v) honey in the reaction mixture increased the Δ*G*_*D*_^25°C^ value from 4400 cal/mol (for FITC-BSA) to 5500 cal/mol and 8400 cal/mol, respectively. The enthalpy change of thermal denaturation of FITC-BSA both in the absence and presence of 10% and 20% (w/v) honey, as determined from the van't Hoff plot (Figure 6), also showed significant increase in the ΔH value from 41800 cal/mol for FITC-BSA to 55000 cal/mol in the presence of 20% (w/v) honey (Table 2), suggesting thermal stabilization of FITC-BSA in the presence of honey. Although an increase in Δ*G*_*D*_^H_2_O^ and Δ*G*_*D*_^25°C^of FITC-BSA was noticed in the presence of honey, the increase in Δ*G*_*D*_^25°C^ was much higher compared to the increase in Δ*G*_*D*_^H_2_O^ obtained in urea denaturation experiments. In other words, the presence of honey in the protein solution offered greater thermal stability to the protein than chemical stability. Such a difference might be attributed to different mechanisms of denaturation as well as different denatured states of the protein, obtained under urea and thermal denaturations [32, 33].

**Figure 7:**
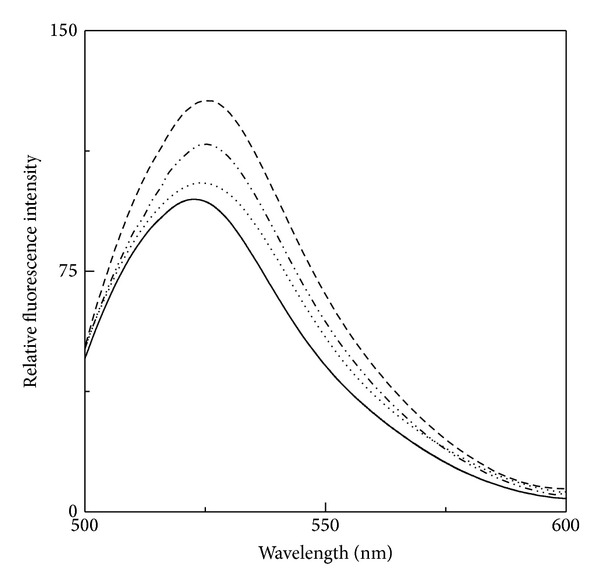
Fluorescence spectra of FITC-BSA at 25°C (—) and 70°C both in the absence (- - -) and presence of 10% (– *·* *·* –) and 20% (*·* *·* *·* *·*) (w/v) honey.

Figure 7 shows the fluorescence spectra of FITC-BSA (at 25°C) and thermal-denatured FITC-BSA at 70°C both in the absence and presence of 10% and 20% (w/v) honey. There was a significant increase (~30%) in the fluorescence intensity at 522 nm along with 2 nm red shift in the 70°C thermal-denatured FITC-BSA, which were suggestive of protein denaturation. The presence of 10% and 20% (w/v) honey in the reaction mixture led to a significant reversal in the fluorescence intensity towards FITC-BSA fluorescence, being more significant in the presence of 20% (w/v) honey. About ~47% and ~84% reversals in the fluorescence intensity at 522 nm were observed in the presence of 10% and 20% (w/v) honey, respectively. These results further supported the protein stabilization effect of honey against thermal denaturation of the protein.

 High sugar content and the presence of almost all amino acids in honey have rendered it to be an effective protein stabilizer as both of these components are osmolytes whose protein stabilizing potential is well known [1, 11–14, 16]. Different mechanisms have been proposed to explain the osmolyte-induced stabilization of proteins under denaturing conditions [11, 34–36]. According to Smith and coworkers [34], addition of osmolytes to aqueous protein solution changes the solvent properties leading to stronger hydrophobic interactions in protein and reduces the hydrogen rupturing potency of vicinity water molecules. On the other hand, both “preferential hydration” and “preferential exclusion” phenomena dominate the osmolyte-induced stabilization mechanism proposed by Timasheff and his group [10, 11, 36]. Moreover, Bolen and Baskakov [35] emphasized the importance of unfavourable solvophobic interactions between osmolyte and polypeptide backbone, which was termed as “osmophobic effect,” as the main contributor towards increasing Gibbs free energy. Overall, these mechanisms have suggested the displacement of equilibrium between protein's native state and denatured state towards the native state in presence of osmolyte, thus stabilizing the protein against denaturing conditions. 

 Due to the presence of different sugars and amino acids in honey, the stabilizing effect observed in the presence of honey would be the additive effect of all the osmolytes present in the honey. As these compounds are known to be present in different honey samples [18], honey samples from different origins are supposed to show the similar protein stabilizing potential. Several earlier reports on osmolyte-induced stabilization of proteins by cosolute mixtures have also suggested the additive effect of the osmolytes [12, 15, 16]. Thus, honey (a bioresource) being a mixture of different osmolytes would have offered greater stabilization than the individual osmolytes. 

 In conclusion, FITC-BSA was found to be stabilized against urea and thermal denaturations by different honey concentrations. Hence, honey can be used as a protein stabilizer in food related industries.

## Figures and Tables

**Table 1:** Characteristics of urea denaturation of FITC-BSA in the absence and presence of different honey concentrations as monitored by FITC fluorescence upon excitation at 495 nm.

Protein sample	Transition			Δ*G*_*D*_^H_2_O^ (cal mol^−1^)
	Start point [M]	Midpoint [M]	End point [M]	
FITC-BSA	4.0	5.7	9.3	3400
FITC-BSA + 10% (w/v) honey	4.2	5.8	9.3	3700
FITC-BSA + 20% (w/v) honey	4.4	6.1	9.3	4400

**Table 2:** Characteristics of thermal denaturation of FITC-BSA in the absence and presence of different honey concentrations as monitored by FITC fluorescence upon excitation at 495 nm.

Protein sample	Transition			Δ*G*_*D*_^25°C^ (cal mol^−1^)	ΔH (cal mol^−1^)
	Start point (°C)	Midpoint (°C)	End point (°C)		
FITC-BSA	45.0	69.3	95.0	4400	41800
FITC-BSA + 10% (w/v) honey	57.5	75.0	95.0	5500	51000
FITC-BSA + 20% (w/v) honey	62.5	82.8	95.0	8400	55000
